# Severe Diffuse Ulcerative Esophagitis Following Treatment with Enfortumab Vedotin and Pembrolizumab in Metastatic Urothelial Carcinoma: A Case Report

**DOI:** 10.3390/reports9030237

**Published:** 2026-07-22

**Authors:** Navanita Biswas, Shoja Rahimian

**Affiliations:** 1 Department of Internal Medicine, Tower Health Reading Hospital, West Reading, PA 19608, USA; 2 Department of Hematology & Oncology, Tower Health Reading Hospital, West Reading, PA 19608, USA; shoja.rahimian@towerhealth.org

**Keywords:** enfortumab vedotin, pembrolizumab, immune-related adverse event, esophagitis, urothelial carcinoma

## Abstract

**Background and Clinical Significance**: Enfortumab vedotin combined with pembrolizumab has emerged as an effective first-line therapy for advanced urothelial carcinoma. While immune checkpoint inhibitors are associated with digestive tract toxicities, upper gastrointestinal involvement such as esophagitis remains rare, and its presentation in combination with enfortumab vedotin is not well characterized. **Case Presentation**: A 72-year-old man with metastatic urothelial carcinoma presented with generalized weakness, poor oral intake, odynophagia, dysphagia, anemia, and systemic symptoms following the second cycle of combination therapy of enfortumab vedotin and pembrolizumab. Endoscopic evaluation revealed diffuse circumferential ulcerative esophagitis involving the entire esophagus, with associated duodenitis. Infectious workup, including Clostridioides difficile, cytomegalovirus, and human immunodeficiency virus testing, was negative, and HSV-1 IgG was positive, consistent with prior exposure rather than active infection; however, tissue-based testing for active HSV infection was not performed. Lower gastrointestinal evaluation demonstrated nonspecific rectal inflammation. The patient was treated with high-dose intravenous corticosteroids (intravenous methylprednisolone 1 mg/kg/day) with rapid clinical improvement within 48–72 h, followed by a steroid taper and supportive care. **Conclusions**: This case represents a severe and diffuse manifestation of esophagitis associated with enfortumab vedotin and pembrolizumab therapy. While immune-mediated esophagitis is rare, the combination of antibody–drug conjugate therapy with immune checkpoint inhibition may contribute to synergistic mucosal injury. Early recognition and prompt initiation of immunosuppressive therapy are critical for favorable outcomes. Clinicians should be aware of severe esophagitis as a potential complication of enfortumab vedotin and pembrolizumab therapy. Timely diagnosis and management with corticosteroids can lead to rapid symptom resolution and may prevent serious complications.

## 1. Introduction and Clinical Significance

Advanced urothelial carcinoma remains a therapeutically challenging malignancy, particularly in patients with metastatic disease. Recent advances in systemic therapy have introduced novel combinations that improve outcomes compared to traditional platinum-based regimens. Among these, the combination of Enfortumab vedotin and Pembrolizumab has emerged as an effective first-line treatment option for patients with locally advanced or metastatic urothelial carcinoma who are ineligible for cisplatin-based chemotherapy [1].

Enfortumab vedotin is an antibody–drug conjugate targeting nectin-4, a protein highly expressed in urothelial carcinoma, delivering the microtubule-disrupting agent monomethyl auristatin E directly to tumor cells. Notably, nectin-4 is also expressed on normal epithelial tissues, including the squamous epithelium of the skin and upper aerodigestive tract, providing a mechanistic basis for on-target, off-tumor mucocutaneous toxicity. Pembrolizumab, a programmed death-1 (PD-1) inhibitor, enhances antitumor immunity by blocking inhibitory signaling pathways that limit T-cell activation. The combination of these agents has demonstrated significant clinical benefit, as evidenced in the EV-103 trial [2] and subsequently confirmed in the phase III EV-302 trial [1], which showed improved overall survival and progression-free survival compared to standard chemotherapy.

Despite its efficacy, this combination therapy is associated with a distinct toxicity profile. Enfortumab vedotin is commonly associated with adverse effects including cutaneous reactions, peripheral neuropathy, and hyperglycemia [2]. In contrast, pembrolizumab is associated with immune-related adverse events affecting multiple organ systems, most commonly involving the gastrointestinal tract, liver, endocrine organs, and skin [3]. Gastrointestinal immune-related toxicities most frequently manifest as colitis, while upper gastrointestinal involvement such as esophagitis is rare but increasingly recognized [3,4].

While esophagitis associated with immune checkpoint inhibitors has been reported in the literature, the occurrence of severe esophageal toxicity in patients receiving combination therapy with enfortumab vedotin and pembrolizumab remains exceedingly uncommon. To our knowledge, only one prior case has described esophagitis specifically in the setting of this combination regimen [5].

Here, we present a case of severe diffuse circumferential ulcerative esophagitis (CTCAE v5.0 Grade 3) in a patient receiving enfortumab vedotin and pembrolizumab for metastatic urothelial carcinoma. This case highlights a rare but potentially life-threatening manifestation of combination therapy-associated gastrointestinal toxicity and underscores the importance of early recognition and management.

## 2. Case Presentation

A 72-year-old man with stage IV high-grade urothelial carcinoma with pulmonary metastases presented shortly after completion of Cycle 2 Day 8 of therapy with generalized weakness, poor oral intake, odynophagia, dysphagia, nausea, vomiting, diarrhea, and fevers up to 102 °F at home. His past medical history was significant for chronic kidney disease stage 3b, hypertension, hyperlipidemia, type 2 diabetes mellitus, prior transient ischemic attack, and hyperuricemia.

His urologic and oncologic history began during hospitalization for acute stroke complicated by acute kidney injury, with serum creatinine rising to approximately 1.4–1.5 mg/dL. Renal and bladder ultrasonography demonstrated a 1.7 × 1.3 cm mineralized density adherent to the bladder wall. Cystoscopy subsequently revealed multiple sessile and flat areas concerning for urothelial malignancy and an approximately 2 cm papillary lesion along the posterior bladder wall. He underwent transurethral resection of the bladder tumor, which demonstrated high-grade T1 urothelial carcinoma with carcinoma in situ and lymphovascular invasion, without muscularis propria invasion. He then underwent upfront radical cystectomy, with final pathology demonstrating high-grade poorly differentiated urothelial carcinoma, pT3aN0M0. Post-procedurally, renal function stabilized with serum creatinine approximately 1.1–1.2 mg/dL. He subsequently received adjuvant nivolumab for approximately four months, after which pulmonary metastatic progression was confirmed by biopsy and therapy was transitioned to enfortumab vedotin plus pembrolizumab. Treatment was administered as follows: Cycle 1 Day 1 included enfortumab vedotin and pembrolizumab 200 mg; Cycle 1 Day 8 included enfortumab vedotin alone; Cycle 2 Day 1 included both enfortumab vedotin and pembrolizumab; and Cycle 2 Day 8 included enfortumab vedotin 90 mg alone. Symptom onset occurred approximately four days after the most recent enfortumab vedotin infusion and 11 days after the second pembrolizumab dose. He denied use of medications commonly associated with pill esophagitis, including bisphosphonates, doxycycline, potassium chloride, nonsteroidal anti-inflammatory drugs, or oral iron supplementation, and had no history of prior thoracic radiation. His home medications included metformin, atorvastatin, and levothyroxine. None of these medications are classically associated with pill-induced esophagitis, and no clinically significant medication interactions were identified that would independently explain the severe diffuse ulcerative esophageal injury.

On admission, he was found to have acute anemia with a hemoglobin decline from a baseline of 11–12 g/dL to 7.0–7.6 g/dL, requiring transfusion of one unit of packed red blood cells. His hospital course was complicated by hypotension, acute hypoxic respiratory failure, and new-onset atrial fibrillation with rapid ventricular response requiring transfer to the medical intensive care unit. These complications were considered multifactorial in the setting of acute anemia, suspected upper gastrointestinal blood loss, poor oral intake, systemic illness, and atrial fibrillation with rapid ventricular response, rather than solely attributable to esophageal bleeding. Computed tomography imaging did not show findings concerning for immune-related pneumonitis, and troponin was within normal limits, making immune-related cardiomyopathy or myocarditis less likely as the primary cause of hemodynamic instability. He was treated with intravenous amiodarone and metoprolol with subsequent stabilization.

**Figure 1:**
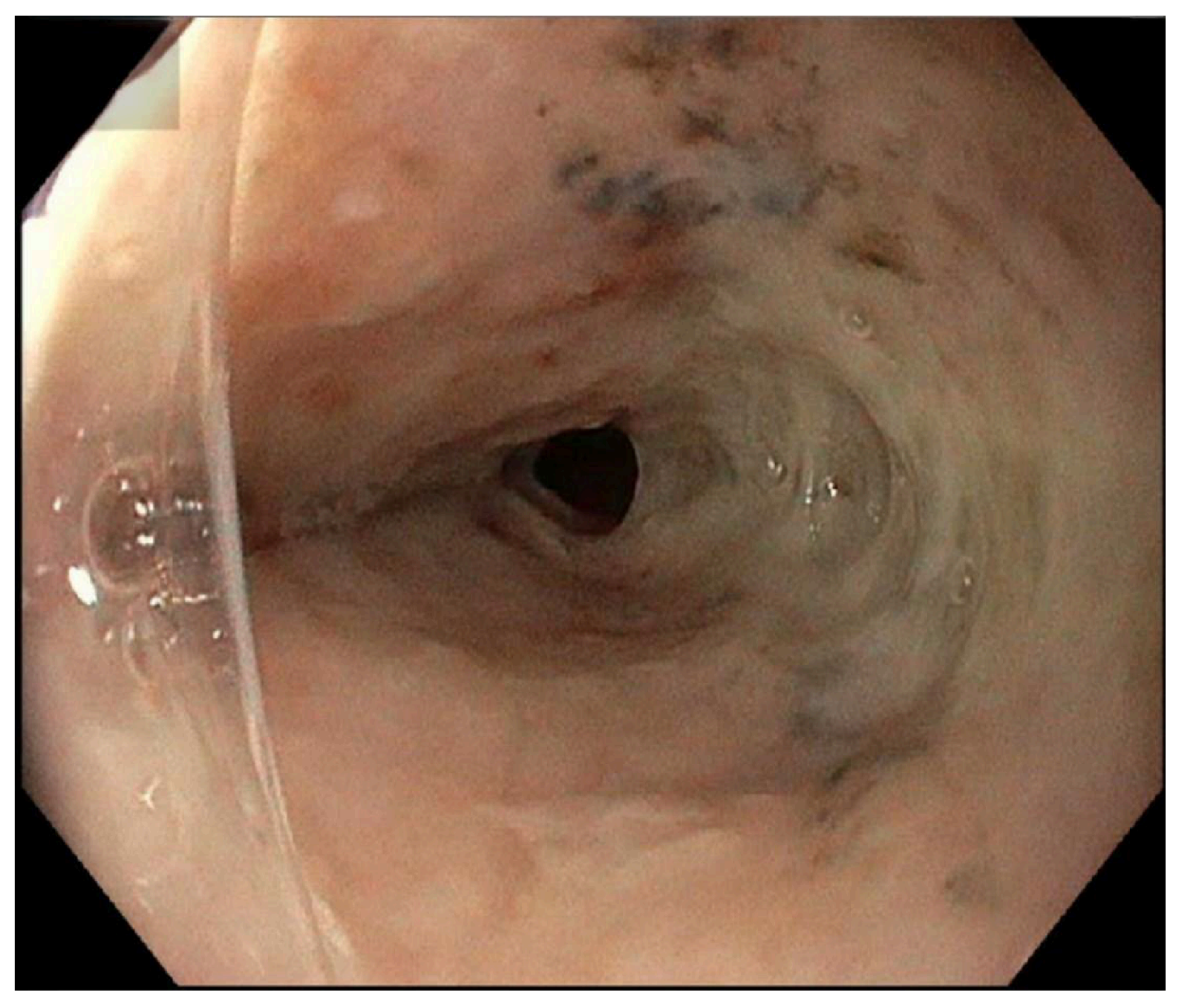
Endoscopic image showing diffuse circumferential ulcerative esophagitis with dark punctate areas concerning for focal mucosal necrosis.

Computed tomography of the abdomen and pelvis showed no evidence of active gastrointestinal bleeding. However, given persistent anemia and concern for melena, upper endoscopic evaluation was performed. Esophagogastroduodenoscopy revealed diffuse ulcerative esophagitis extending from 18 cm to 38 cm from the incisors, with pinpoint red and black spots concerning for focal mucosal necrosis (Figure 1). The esophagitis was classified as CTCAE v5.0 Grade 3 based on hospitalization, inability to maintain adequate oral intake, and need for systemic corticosteroid therapy, rather than direct attribution of all ICU-level complications to esophageal injury. Additional findings included probable 3 cm Barrett’s esophagus, a 3 cm hiatal hernia, and a 1 cm villous-appearing polyp at the gastroesophageal junction/hiatal hernia region, which was biopsied and showed a fundic gland polyp without dysplasia. No additional gastric mucosal abnormalities were documented in the EGD report other than the hiatal hernia and gastroesophageal junction/hiatal hernia polyp. Severe duodenitis was also noted, along with a duodenal mucosal nodule/polyp in the proximal second portion of the duodenum that was not biopsied because of the clinical indication and procedural risk. Biopsies of the diffusely ulcerated esophageal mucosa were deferred because of severe ulceration, mucosal friability, and concern for bleeding or perforation risk.

Given the severity and diffuse nature of the esophageal injury, concern was raised for immune-mediated mucosal toxicity versus treatment-related injury from enfortumab vedotin and/or pembrolizumab. The severe esophageal injury was considered a major contributor to his odynophagia, poor oral intake, and suspected upper gastrointestinal blood loss; however, definitive histopathologic confirmation was not available.

**Figure 2:**
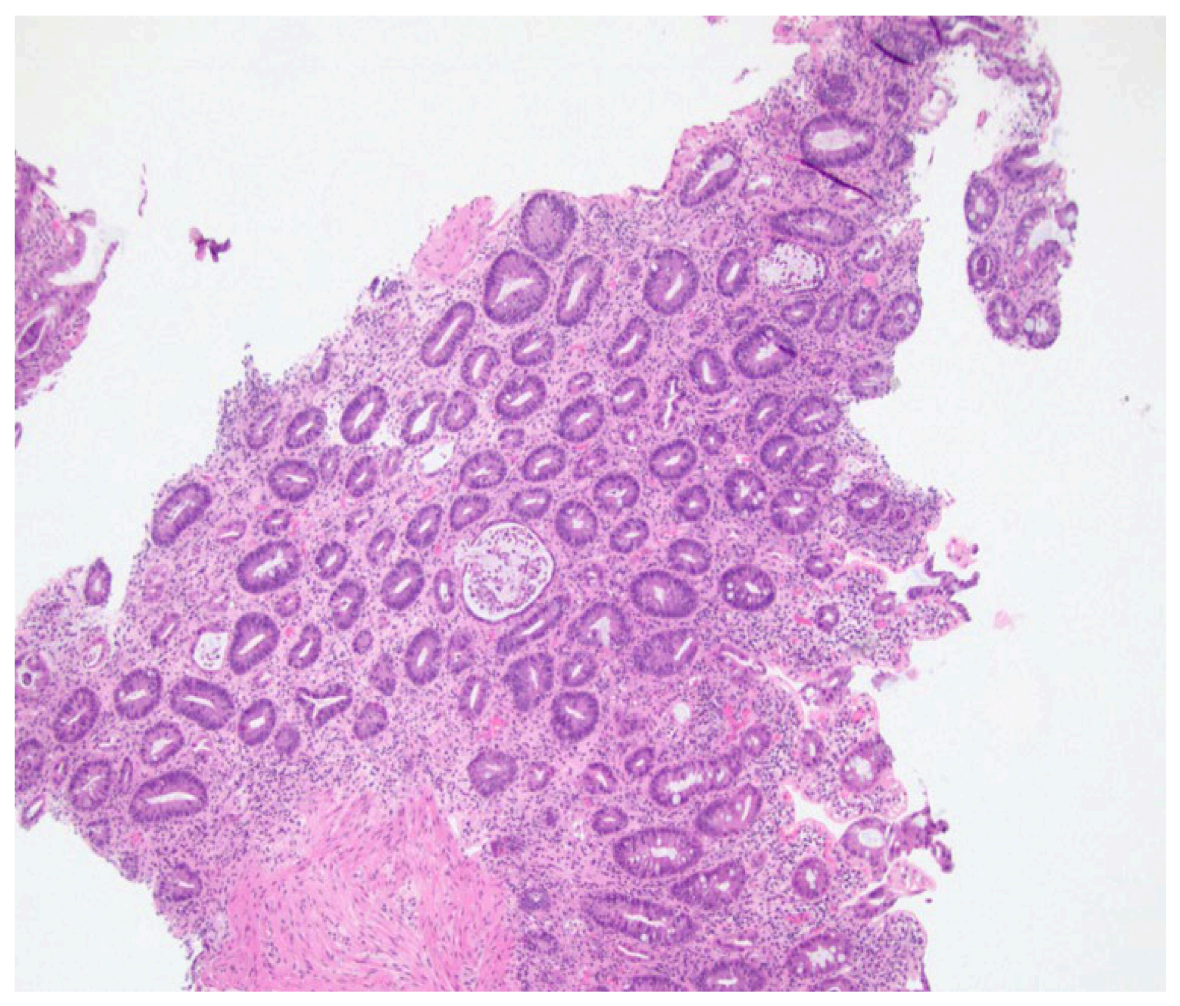
Photomicrograph of rectal mucosa showing cryptitis and crypt abscesses (hematoxylin and eosin stain; 10× objective magnification).

Flexible sigmoidoscopy demonstrated superficial rectal ulcerations without active bleeding and mild sigmoid diverticulosis. Rectal biopsies showed acute cryptitis, crypt abscess formation, ulceration, lamina propria hyalinization, and necrosis (Figure 2). These findings were nonspecific and raised a differential diagnosis including ischemic injury, stercoral colitis, and immune checkpoint inhibitor-associated colitis; however, the limited rectal distribution was not entirely typical for diffuse immune-mediated colitis.

Infectious evaluation was negative, including stool Clostridioides difficile testing and cytomegalovirus and human immunodeficiency virus testing. Stool studies demonstrated positive lactoferrin, consistent with active intestinal inflammation, while fecal calprotectin was negative. HSV-1 IgG was positive (7.96), consistent with prior exposure; however, tissue-based testing for active herpes simplex virus infection was not performed (see Section 4).

Overall, in the absence of an identifiable infectious etiology and in the setting of immune checkpoint inhibitor therapy, the clinical and endoscopic findings were most consistent with severe diffuse ulcerative esophagitis, likely immune-related or drug-induced in etiology, with concurrent but less clearly defined lower gastrointestinal inflammation.

**Figure 3:**
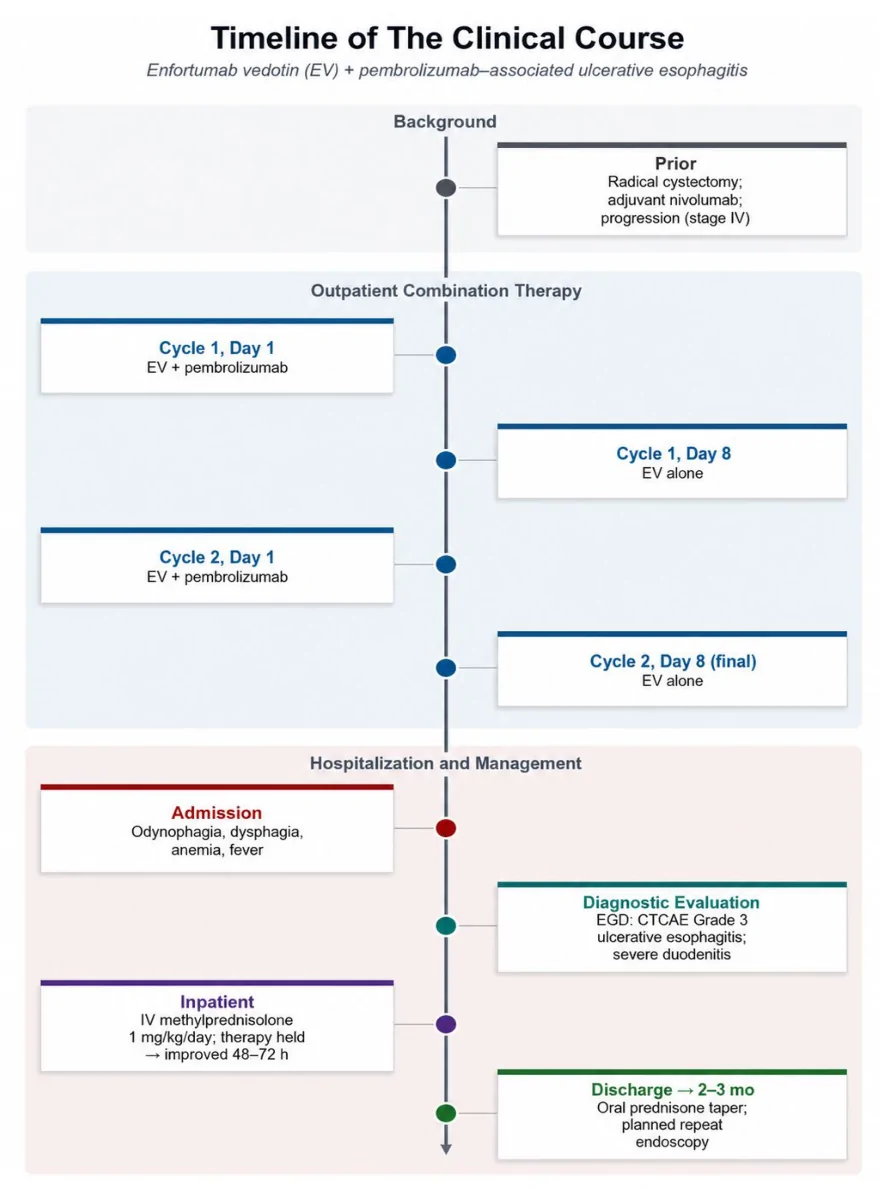
Timeline summarizing the prior oncologic history, enfortumab vedotin (EV) plus pembrolizumab treatment, symptom onset, diagnostic evaluation, inpatient management, and planned follow-up. Abbreviations: CTCAE, Common Terminology Criteria for Adverse Events; EGD, esophagogastroduodenoscopy.

The patient was initiated on high-dose intravenous methylprednisolone at 1 mg/kg/day, and both enfortumab vedotin and pembrolizumab were held. He demonstrated rapid clinical improvement within 48–72 h, with marked reduction in odynophagia and dysphagia, improved oral intake, stabilization of hemoglobin levels, and resolution of systemic symptoms. The patient was transitioned to an oral prednisone taper starting at 50 mg daily, with a planned gradual taper over six weeks. He was maintained on proton pump inhibitor therapy with pantoprazole 40 mg twice daily. A repeat endoscopic evaluation was planned in 2–3 months to assess mucosal healing. At discharge, the patient remained clinically stable without further transfusion requirements, and outpatient oncology follow-up was arranged to determine the feasibility of future systemic therapy. On additional chart review at the time of manuscript revision, repeat esophagogastroduodenoscopy had not yet been performed, and enfortumab vedotin plus pembrolizumab remained on hold pending further clinical recovery and multidisciplinary oncology evaluation. Therefore, treatment rechallenge had not occurred, and recurrence of esophageal toxicity after rechallenge could not be assessed. The overall clinical course, from initiation of combination therapy through symptom onset, diagnostic evaluation, management, and planned follow-up, is summarized in Figure 3.

## 3. Discussion

Immune checkpoint inhibitor (ICI) therapy has transformed the management of advanced malignancies, including urothelial carcinoma, through durable responses and improved survival outcomes. However, ICIs are associated with immune-related adverse events (irAEs) affecting multiple organ systems, most commonly the gastrointestinal tract. While colitis is the predominant manifestation, upper gastrointestinal involvement, including esophagitis, remains uncommon.

ICI-related esophagitis has been described in a limited number of case reports and small institutional series. Reported clinical presentations include odynophagia, dysphagia, and poor oral intake, often associated with endoscopic findings of erosions or ulcerations. Severe immune-mediated mucositis and esophagitis have been reported in patients receiving pembrolizumab, supporting the potential for upper gastrointestinal irAEs, albeit rarely [6,7,8].

In contrast, a large retrospective cohort from a tertiary cancer center demonstrated that immune checkpoint inhibitor-related esophagitis is identified in approximately 3% of patients undergoing endoscopic evaluation, with most cases showing concurrent gastric or duodenal involvement rather than isolated esophageal disease [9]. These findings highlight that true isolated esophageal involvement is uncommon and often occurs in the setting of more diffuse upper gastrointestinal inflammation. The presence of severe duodenitis and nonspecific rectal inflammatory changes suggests that the toxicity in this case may not have been limited to the esophagus. Rather, the findings support a multifocal gastrointestinal mucosal injury pattern, with the most severe and clinically prominent manifestation involving diffuse circumferential ulcerative esophagitis. Although the rectal biopsy findings raised consideration of immune checkpoint inhibitor-associated colitis, the limited rectal distribution and nonspecific histologic features precluded definitive attribution. Therefore, the overall clinical picture was interpreted as probable treatment-related upper gastrointestinal mucosal injury with concurrent but less clearly defined lower gastrointestinal inflammation.

Enfortumab vedotin, an antibody–drug conjugate targeting nectin-4, is associated with a distinct toxicity profile characterized primarily by dermatologic reactions, peripheral neuropathy, and metabolic disturbances, with gastrointestinal mucosal injury being infrequently reported [1,2]. In a FAERS-based post-marketing pharmacovigilance study, Yu et al. identified dermatologic toxicity and peripheral neuropathy as major adverse events associated with enfortumab vedotin, with additional signals for gastrointestinal, hepatic, and pulmonary events [10].

The combination of enfortumab vedotin with pembrolizumab has demonstrated significant clinical efficacy in advanced urothelial carcinoma, as established in clinical trials, and is now considered a standard first-line therapeutic option. However, its toxicity profile in real-world settings is still being defined.

Severe esophageal injury associated with enfortumab vedotin plus pembrolizumab has been reported only in isolated case reports, suggesting a potential but rare association [5]. In the previously published case, severe esophageal ulceration occurred during therapy, and the patient was managed with immunosuppression and treatment interruption, with consideration of rechallenge depending on clinical recovery. In contrast, our patient presented with diffuse circumferential ulcerative esophagitis (CTCAE v5.0 Grade 3) involving the entire esophagus, with concern for mucosal necrosis and suspected upper gastrointestinal blood loss, representing a more extensive and severe phenotype of upper gastrointestinal injury.

The patient’s comorbidities may also have contributed to baseline susceptibility to esophageal injury. In particular, diabetes mellitus has been associated with esophageal dysmotility, delayed gastric emptying, gastroesophageal reflux, impaired mucosal healing, and increased susceptibility to esophageal inflammation [11,12,13]. Chronic kidney disease may also contribute to impaired mucosal healing, altered host defenses, and increased physiologic vulnerability during acute illness. Therefore, diabetes mellitus and chronic kidney disease may have represented predisposing factors in this patient. Nevertheless, the close temporal relationship with enfortumab vedotin plus pembrolizumab, the diffuse circumferential pattern of ulceration, associated duodenal and rectal inflammatory findings, and rapid clinical improvement following treatment interruption and systemic corticosteroid therapy support probable treatment-related mucosal injury occurring in a susceptible host.

The probable Barrett’s esophagus and hiatal hernia identified during endoscopy may also represent pre-existing reflux-related abnormalities that increased susceptibility to esophageal injury. However, because no prior esophagogastroduodenoscopy was available, it is unknown whether these findings predated oncologic therapy. Although underlying reflux-related disease may have predisposed the patient to esophageal injury, it is unlikely to fully explain the abrupt onset, diffuse circumferential ulcerative pattern, associated duodenitis and rectal inflammatory changes, and rapid clinical improvement following corticosteroid therapy.

The patient’s prior exposure to adjuvant nivolumab may also be clinically relevant. Delayed immune-related adverse events can occur after discontinuation of immune checkpoint inhibitor therapy, and prior PD-1 inhibitor exposure may theoretically influence susceptibility to subsequent immune-mediated toxicity. In this case, nivolumab had been discontinued approximately two months before the current hospitalization. Because symptoms developed shortly after initiation of enfortumab vedotin plus pembrolizumab, the current presentation was considered most temporally associated with the combination regimen. However, the specific contribution of prior nivolumab exposure cannot be determined. Immune checkpoint inhibitor-associated esophagitis does not have a single pathognomonic clinical, endoscopic, or histologic feature. Reported cases commonly present with odynophagia, dysphagia, poor oral intake, and endoscopic evidence of erosive or ulcerative mucosal injury, sometimes with concurrent gastric, duodenal, or lower gastrointestinal inflammation. In the present case, the diffuse circumferential ulcerative pattern, associated duodenitis and rectal inflammatory findings, close temporal relationship with therapy, and rapid corticosteroid responsiveness supported probable treatment-related mucosal injury after exclusion of common alternative triggers.

The pathophysiology of severe esophageal injury in this setting is likely multifactorial. Pembrolizumab disrupts peripheral immune tolerance through PD-1 blockade, which can result in T-cell-mediated epithelial injury at mucosal sites [14]. Enfortumab vedotin may exacerbate mucosal vulnerability through cytotoxic payload delivery and epithelial barrier disruption. Co-administration may therefore produce synergistic mucosal injury through complementary mechanisms: ICI-driven loss of tolerance and ADC-mediated epithelial cytotoxicity.

Management of ICI-related esophagitis is primarily based on interruption of the suspected offending therapy, supportive care, and systemic corticosteroids for clinically significant immune-related toxicity. Current ASCO guidelines recommend holding immune checkpoint inhibitor therapy and initiating systemic corticosteroids at a 1–2 mg/kg/day prednisone equivalent for grade 3 immune-related adverse events, followed by a gradual taper over at least 4–6 weeks [15]. In more severe or steroid-refractory cases, additional immunosuppressive agents may be required. In our patient, high-dose intravenous corticosteroids resulted in clinical improvement, with stabilization of hemoglobin levels and improvement in oral intake, supporting an immune-mediated component to the injury. Proton pump inhibitor therapy and supportive care were also administered.

Beyond acute management, severe toxicity in metastatic disease raises the question of long-term treatment strategy. While therapy may be considered for resumption after grade 3 events that resolve, permanent discontinuation is generally recommended for grade 4 or severe recurrent toxicities [15]. Reported recurrence rates following immune checkpoint inhibitor rechallenge range from approximately 29% to 43% [16,17]. In this case, the decision is further complicated by uncertainty regarding the responsible agent, as both enfortumab vedotin and pembrolizumab may have contributed. These risks must be weighed against the risk of disease progression if effective therapy is withheld. Such decisions warrant multidisciplinary oncology evaluation, with documentation of mucosal healing before any rechallenge when feasible.

A key distinction between our case and the previously reported case of enfortumab vedotin plus pembrolizumab-associated esophagitis is the absence of a follow-up endoscopic reassessment in our patient at the time of reporting. Additionally, the prior case described consideration of treatment rechallenge, whereas in our case, therapy remains on hold pending clinical recovery and multidisciplinary oncologic evaluation. This represents a limitation in assessing mucosal healing and long-term outcomes following severe injury.

## 4. Limitations

This report has several limitations. First, biopsies of the diffusely ulcerated esophageal mucosa were not performed at the time of initial endoscopy because of concern for procedural bleeding and perforation risk in the setting of severe ulceration and mucosal friability, limiting histopathologic confirmation of the underlying esophagitis. Second, targeted viral evaluation from tissue, including herpes simplex virus and cytomegalovirus testing by immunohistochemistry or polymerase chain reaction, as well as plasma cytomegalovirus PCR, was not obtained, which may limit complete exclusion of viral etiologies. Third, despite additional chart review at the time of manuscript revision, follow-up endoscopic reassessment had not yet been performed, restricting evaluation of mucosal healing and long-term outcomes. Finally, enfortumab vedotin plus pembrolizumab remained on hold pending further clinical recovery and multidisciplinary oncology evaluation; therefore, treatment rechallenge had not occurred, and recurrence of esophageal toxicity after rechallenge could not be assessed.

Overall, this case highlights a rare but clinically significant manifestation of severe diffuse ulcerative esophagitis associated with combination enfortumab vedotin and pembrolizumab therapy. It expands the emerging spectrum of upper gastrointestinal toxicity associated with novel systemic therapies in urothelial carcinoma and underscores the importance of early recognition and prompt immunosuppressive management.

## 5. Conclusions

This case highlights a rare but severe presentation of diffuse ulcerative esophagitis associated with combination enfortumab vedotin and pembrolizumab therapy. Although upper gastrointestinal immune-related adverse events are uncommon, clinicians should maintain a high index of suspicion in patients presenting with odynophagia, dysphagia, or unexplained anemia while on this regimen. Early endoscopic evaluation and prompt initiation of corticosteroid therapy may lead to rapid clinical improvement and prevent further complications. As the use of this combination continues to expand, recognition of atypical and severe gastrointestinal toxicities is essential to guide timely management and optimize patient outcomes.

## Data Availability

The original data presented in the study are included in the article, further inquiries can be directed to the corresponding author.
